# Provision of Enteral Nutrition in a Critically Ill Patient Requiring Multiple Prone Ventilation Sessions

**DOI:** 10.7759/cureus.82101

**Published:** 2025-04-11

**Authors:** Reynald Jaenelle A Manlungat, Dore Chikkahanasoge Ananthegowda, Anwar Mohd. Faleh Qudaisat, Jadulluh Mohammad Abdulluh Alghazo, Virendra Pratap Chaudhary, Sibusiso Reuben Katama, Mohammad Ghassan Ragheb Abdelbaset

**Affiliations:** 1 Dietetics and Nutrition, Hamad Medical Corporation, Doha, QAT; 2 Critical Care, Hamad Medical Corporation, Doha, QAT

**Keywords:** acute respiratory distress syndrome, critical care, enteral nutrition, feed intolerance, prone ventilation

## Abstract

Timely and adequate nutrition support in critical care is necessary to prevent metabolic deterioration and preserve lean body mass. However, providing enteral nutrition during prone ventilation carries certain risks. The potential for gastrointestinal intolerance and ventilator-associated pneumonia is a concern in this context. Furthermore, healthcare providers are often hesitant to start or continue enteral nutrition for patients in prone ventilation due to fears of gastrointestinal complications. Here we describe a case involving a 53-year-old male patient admitted to the critical care unit, diagnosed with acute respiratory distress syndrome (ARDS), requiring mechanical ventilation and multiple rounds of prone ventilation. Enteral feeding was initiated and increased to a maximum rate of 65 ml/hour, allowing the patient to meet his energy and protein needs while in prone ventilation. The feeding rate was well tolerated, with no adverse effects reported. Complete nutritional requirements could be satisfied even amid the demands of multiple prone ventilation sessions in a critically ill patient.

## Introduction

Prone ventilation has emerged as a proven therapeutic option to enhance oxygenation in acute respiratory distress syndrome (ARDS) [1]. This technique involves shifting the patient from the supine to the prone position, potentially improving survival rates and reducing the duration of mechanical ventilation by modifying regional transpulmonary pressure and reducing lung aeration heterogeneity [2]. These effects contribute to less lung trauma and improved gas exchange in ARDS patients [2].

ARDS is characterized by an acute, inflammatory lung injury that may lead to life-threatening conditions in critically ill patients, marked by sudden hypoxemia and bilateral pulmonary edema due to increased alveolocapillary permeability [2,3]. The extended hospital stays associated with ARDS can result in significant weight loss, decreased muscle function, functional impairment, and a mortality rate that may reach up to 40% [2,3].

Nutrition therapy plays a vital role in managing critically ill patients. ARDS can cause a 20% increase in daily energy expenditure alongside heightened protein catabolism and pro-inflammatory responses [4]. In mechanically ventilated patients, early nutritional intervention can strengthen gastrointestinal mucosa, boost immune function, reduce complications, improve patient outcomes, shorten hospital stays, and decrease mortality and costs [5,6]. Recent guidelines recommend initiating early nutrition within 48 hours for patients on prone ventilation unless contraindications exist [6]. However, direct international guidance or local protocols on enteral nutrition for this patient group are lacking.

Multiple challenges exist when providing enteral nutrition during prone ventilation, including increased intra-abdominal pressure, a heightened risk of gastroesophageal reflux, feeding intolerance, and ventilator-associated pneumonia [7]. There are also concerns regarding aspiration risk due to gastrointestinal intolerance (e.g., nausea, vomiting, and regurgitation), which can affect healthcare providers' decisions about continuing enteral nutrition or achieving the necessary feeding rates and energy levels for prone patients [7,8].

High-quality studies and case reports assessing the feasibility of enteral nutrition during prone ventilation are limited. Some research indicates that early enteral nutrition can lead to vomiting, safety concerns, and reports of poor tolerability [5,9]. A case review by Panter and Caffrey documented a patient with ARDS requiring prone ventilation who reached a feeding rate of 100 ml/hour without evidence of gastroesophageal reflux and successfully met his nutritional requirements [10]. However, literature addressing enteral nutrition in critically ill patients requiring multiple prone ventilation sessions is sparse and inconsistent. This case report aims to explore the feasibility of enteral nutrition in critically ill patients undergoing multiple prone ventilation sessions.

This article was accepted and presented as a conference e-abstract at the 2025 Middle Eastern Alliance for Parenteral and Enteral Nutrition Congress on April 25-26, 2025. Institutional Review Board approval was secured from the ethical committee at the medical research center of Hamad Medical Corporation, Qatar. 

## Case presentation

A 53-year-old male patient with no known comorbidities presented to the emergency unit with fever, cough, and shortness of breath for four days. He was transferred to the critical care unit due to increasing oxygen requirements and later diagnosed with severe community-acquired pneumonia caused by influenza A, H1N1 positive, and severe ARDS.

The patient's condition was deteriorating despite the use of high-flow nasal cannula and noninvasive ventilation, failing to improve with oxygen support. The medical team decided to sedate and intubate the patient due to persistent tachypnea and desaturation. The patient was placed in prone ventilation for 16 hours a day. In total, he underwent prone ventilation seven times during his stay in the critical care unit. During this, the patient was positioned in a 20-degree reverse Trendelenburg position.

Nutrition intervention

The patient was referred to the dietetics service for a nutritional assessment. Since he was intubated and sedated, a detailed dietary history could not be obtained. There were no reports of gastric intolerance, such as vomiting, abdominal distention, or diarrhea, during his stay in the emergency unit. However, it was noted that the patient's oral food intake was poor prior to admission to the critical care unit, which may be linked to a loss of appetite and ongoing respiratory distress.

Enteral nutrition commenced within 24 hours of admission per the physician's instructions. The target energy and protein requirements were 1700 kcal/day (25 kcal/kg) and 82 gm/day (1.2 g/kg/day), respectively, calculated based on the patient's weight (68 kg) and guideline recommendations [11]. The feed was delivered via nasogastric tube using a high protein formula with a concentration of 1.28 kcal/ml and a feeding rate of 40 ml/hour over 20 hours of continuous feeding.

During the first two sessions of prone ventilation, the assigned nurse reduced the feeding rate to 20 ml/hour and 35 ml/hour, respectively, due to concerns about vomiting and increased gastric residual volume if the rate were increased further. If not corrected, this practice could compromise the delivery of sufficient energy and protein to the patient.

During the daily medical rounds, the multidisciplinary team agreed not to decrease the feeding rate if no gastrointestinal intolerance was observed throughout feeding in prone ventilation. This approach would ensure that the patient’s energy and protein needs were adequately met. To achieve these requirements, the feeding rate was gradually increased to the goal rate in subsequent prone ventilation sessions.

The feeding rate was gradually raised to 60 ml/hour in the third prone ventilation session. From the fourth to the seventh sessions, the rate was escalated to 65 ml/hour to meet the patient's energy and protein requirements, as noted in Table 1.

**Table 1:** Feed rate progression vs percentage (%) of energy and protein achieved during prone ventilation

	Prone 1	Prone 2	Prone 3	Prone 4	Prone 5	Prone 6	Prone 7
Feed Rate (ml/hour)	20	35	60	65	65	65	65
% energy achieved	31%	54%	93%	100%	100%	100%	100%
% protein achieved	31%	56%	91%	100%	100%	100%	100%

The assigned nurse and dietitian monitored for signs of feeding intolerance during follow-up visits and multidisciplinary team rounds with physicians. There were no reported gastrointestinal intolerances (nausea, vomiting, or regurgitation), and the recorded gastric residual volume was minimal. Furthermore, there was no report of prokinetics use during the patient's stay in critical care unit. After an improvement in health status, the patient was extubated and transitioned to oral feeding as recommended by a speech-language pathologist. The patient was then transferred to the medical floor for further management and rehabilitation.

## Discussion

This case report highlights a patient who received enteral nutrition during multiple prone ventilation sessions in the critical care unit. Few studies specifically report on providing enteral nutrition during prone ventilation. Some studies noted feeding intolerance, while others indicated that any observed intolerance was comparable to that in a supine position [5,7].

The high usage of sedation, occasional use of steroids, and paralysis increase the risk of developing critical illness neuropathy and myopathy, which can predispose to weaning failure. Additionally, inadequate nutrition may lead to further myopathy, prolonged ventilator days, and longer hospital stays [12]. 

The inability to provide adequate energy and protein in critically ill patients is associated with a higher number of complications and infections, extended mechanical ventilation duration, longer hospital stays, and consequently increased costs and mortality rates [4,5]. Conversely, proper nutrition can enhance immunity, reduce complications, improve patient outcomes, decrease the length of stay and ventilator days, and lower hospital costs [5,6].

During prone ventilation, the patient successfully tolerated a maximum feeding rate of 65 ml/hour, fulfilling the energy and protein requirements. No significant gastrointestinal intolerance was observed while providing enteral nutrition, similar to findings in other studies [7,10]. The patient managed to meet his nutritional needs in the critical care unit throughout multiple prone ventilation sessions.

## Conclusions

We report on a critically ill patient diagnosed with ARDS who required enteral nutrition during multiple prone ventilation sessions. This case report illustrates that providing enteral nutrition to patients requiring multiple prone ventilation sessions did not result in gastrointestinal intolerance or increased gastric residual volume. Full nutritional requirements were successfully met, even during the demands of multiple prone ventilation sessions in a critically ill patient. Further larger and higher-quality studies are needed to evaluate the feasibility of providing enteral nutrition during multiple sessions of prone ventilation in critically ill patients.
